# Factors that facilitate recognition and management of domestic violence by primary care physicians in a Chinese context - a mixed methods study in Hong Kong

**DOI:** 10.1186/s12875-020-01228-4

**Published:** 2020-07-30

**Authors:** Tai Pong Lam, Hoi Yan Chan, Leon Piterman, Mei Wa Wong, Kai Sing Sun, Kwok Fai Lam, Tak Hon Chan, Wu Dan, Agnes Tiwari

**Affiliations:** 1 Department of Family Medicine and Primary Care, The University of Hong Kong, HongKong, China; 2 Department of General Practice, Monash University, Melbourne, Australia; 3 Department of Statistics and Actuarial Science, The University of Hong Kong, HongKong, China; 4 Centre for Quantitative Medicine, Duke-NUS Medical School, Singapore City, Singapore; 5 Faculty of Infectious and Tropical Diseases, London School of Hygiene and Tropical Medicine, London, UK; 6 School of Nursing, Hong Kong Sanatorium & Hospital, HongKong, China

**Keywords:** Chinese, Domestic violence, Facilitators, Focus groups, Primary care physicians, Survey

## Abstract

**Background:**

Domestic violence is common in the community. Many of its victims present to primary care physicians (PCPs) but are not being recognized and managed. The barriers, with specific reference to a Chinese cultural context, were investigated earlier. This paper explored the factors which facilitated the process of recognizing and managing suspected cases of domestic violence by PCPs in Hong Kong.

**Methods:**

Four focus group interviews were conducted to explore in-depth the experiences of PCPs in recognition, management and referral of domestic violence cases from which facilitators were identified. The relevant themes were then investigated in a questionnaire survey with 504 PCPs working in public and private sectors.

**Results:**

The focus group participants emphasized mood symptoms as useful indicators for probable abuse and continuity of care was important to unmask issues of domestic violence. The top facilitators perceived by the respondents of the survey included: a trusting doctor-patient relationship (99.8%), good communication skills (99.0%), patients’ unexplained bruises (96.3%), medical history (94.6%), and mood symptoms (94.4%). Further, the survey found that PCPs with longer years of practice, a medical degree obtained from Western countries, and postgraduate training in family counselling or psychological medicine perceived more facilitators in managing domestic violence.

**Conclusions:**

Without a local screening policy and training protocol to manage domestic violence, PCPs regarded their skills in mental healthcare and good relationships with patients as the key facilitators. While training in mental health care helps PCPs manage domestic violence, a specific protocol emphasizing medical-social collaboration is anticipated to facilitate them to take a more proactive and effective stance from screening to management.

## Background

Studies in Hong Kong showed that the prevalence of domestic violence directed against women ranged from 10.4 to 15.7% [1, 2]. These figures are lower than the global prevalence of around 25% [3], including that in the U.S. [4] Domestic violence, also known as intimate partner violence, refers to any behavior within an intimate relationship that causes physical, emotional and sexual harm to the spouse/partner [5, 6]. The vast majority of victims were reported to be women [7–9]. Apart from physical injuries, Chinese studies found high prevalence of severe depression, post-traumatic stress disorder and suicidal behaviors among victims of domestic violence [10–12].

To tackle domestic violence in the community, different national and international frameworks and training resources have been developed to provide guidance and references for primary care physicians (PCPs) [13, 14]. A systematic review identified four common components for domestic violence screening programs, including institutional support, effective screening protocols, initial and ongoing training, and immediate access or referrals to support services. These comprehensive programs led to significantly increased rates of domestic violence screening, identification and disclosure [15]. Amongst the program components, institutional support was considered essential for a comprehensive approach, which required integration and linkage with community resources for health and social care [15].

Providing first-contact care to patients in the community, PCPs have good opportunities to recognize patients who suffer from domestic violence [16], such as patients present with unexplained physical injury, bruising, chronic fatigue, anxiety, depression, insomnia or undifferentiated somatic symptoms. Interviews of PCPs in New Zealand found that cues such as patients’ frequent attendance with ongoing symptoms made them consider the possibility of domestic violence. Besides, PCPs also highlighted the importance of effective collaboration with other practitioners and nurses as well as teachers, police and other agencies for identification and management of domestic violence [16].

Social workers of community agencies play an important role in managing domestic violence cases. A collaborative model has been developed in South Africa in which social workers provide legal and psychosocial support to patients in the community [17]. In Hong Kong, various services for people suffering from domestic violence are provided by the Family and Child Protective Services Units of the Social Welfare Department [18]. The victims can either approach the Units directly or through referrals. The Units also have outreach services, and will render housing, legal and psychological support if required. Medical social workers at special out-patient clinics and public hospitals will also take care of domestic violence cases upon referral by doctors or the police [19]. Furthermore, different non-governmental organizations such as Family Crisis Support Centre and Harmony House have 24-h availability for contact [20]. Nonetheless, as many patients are reluctant to seek help for domestic violence, it is unlikely that they would access the services if they have not been recognized by the health professionals [21].

This study is part of a larger project, which aimed to investigate the barriers and facilitators of PCPs to managing domestic violence in Hong Kong. The findings on the barriers have been reported elsewhere [21]. Four main factors were found, including worries about intervention on domestic issues, lack of guidelines and referral services, limited skills and time constraint of PCPs, and patients’ reluctance in disclosure. Having identified the barriers and Chinese cultural factors, this paper aims to explore the other side of the story. The facilitators and examples of successfully managed cases will be useful for medical educators and policy makers to design training programs for PCPs, and collaboration strategies with other health service providers to manage domestic violence, particularly in a Chinese context.

## Methods

A combined qualitative and quantitative approach was adopted for this study. Ethics approval was obtained from the local Institutional Review Board of The University of Hong Kong/Hospital Authority Hong Kong West Cluster (UW 16–2077).

### Qualitative approach

We started with focus group interviews to explore opinions of the PCPs on the study topic. Similar to the US practice, a PCP in Hong Kong can be a family physician or general practitioner, as well as general pediatrician, internal medicine physician, or a physician in some other fields practicing as a PCP to manage a broad range of common illnesses in the community. The health care system in Hong Kong has a mixed mode of public-private financing [22]. About 75% of primary care services are provided by the private sector (majority are solo practitioners or group practice in clinics), and the rest by the heavily subsidized government general outpatient clinics. The attenders of the public clinics, however, do not have the choice of a regular PCP unless special follow-up consultations are arranged. We purposively recruited PCPs from both public and private clinics with a wide range of characteristics and experiences including years of practice, gender, practice setting, and training background in mental health. Honorary teachers of the Department of Family Medicine and Primary Care of The University of Hong Kong were contacted to recommend participants for the interviews based on their professional network. Invitation letters were sent to the PCPs and followed up by telephone contacts. We conducted four focus group interviews of seven to 10 participants each. There were two focus groups for PCPs working in the public sector and two other groups for those in private practice. Questions on participants’ views towards the barriers and facilitators to managing domestic violence were raised and discussed in the interviews. We aimed to avoid presumption of attitudes of the participants who were encouraged to share their opinions freely. An interview guide developed by the research team was used to ensure relevant questions were covered (Table 1).

**Table 1:** Focus group interview guide

1. What symptoms or signs may enable you to recognize patients encountering domestic violence (intimate partner violence)? Is there any real case that you can share? 2. After recognizing these patients, how do you manage them? Any concerns or difficulties? How do you solve them? 3. Would you refer the patients to other professionals like nurses and social workers? Do the patients follow your advice? 4. How would you evaluate the present collaboration among health professionals in offering help to these patients? Do you have enough support? How can it be strengthened?	

Two experienced facilitators led the 90-min interviews in Cantonese (the local dialect). The interviews were audio-taped and then transcribed verbatim. Using the content analysis approach described by Hsieh and Shannon [23], coding categories were inductively derived from the text data. The data were coded independently by two investigators experienced in qualitative research. Themes were marked manually alongside the coded sentences/paragraphs. The coding consistency between the two sets was checked and the majority of the codes were consistent. Inconsistencies were resolved by discussion between the two investigators to reach agreement for common themes. The key themes identified from the focus group findings were incorporated into the survey questionnaire.

### Quantitative approach

#### Sample

The total number of PCPs is around 6000 in Hong Kong but there is currently no official published list of PCPs. To set a clear sampling frame for the survey, we selected all 1515 members from the Hong Kong College of Family Physicians (HKCFP) as they provided the most readily available list of PCPs. The College members were reached with the help of the HKCFP and a total of three rounds of invitations were sent.

#### Questionnaire

The questionnaire itself was anonymous but coded with a unique reference number to identify the respondent for subsequent rounds of reminders. The code was known to one research assistant only and not available to members of the research team. The questionnaire was posted with an invitation letter which asked for consent of the subject to fill in the questionnaire. Using an exploratory sequential design of mixed methods [24], the questionnaire was designed based on the themes identified from the qualitative phase, with a review of relevant literature and comments from research team members. Details of the questionnaire design and pilot testing were reported elsewhere [21].

#### Statistical analysis

The quantitative data were analyzed using JMP (Release 10.0.0). We used frequencies and percentages to summarize the responses on the facilitator items. One-way ANOVA was carried out to determine the association of PCPs’ background characteristics with the facilitators. A *p*-value < 0.05 was considered statistically significant.

## Results

### Focus groups

Four focus groups comprising 26 PCPs from private and public sectors were held between September and November 2017. Among them, 57.7% were male and 42.3% were female. The mean years after graduation from medical school was 18.8. The major themes of the PCPs’ perceived facilitators and ways to recognize and manage domestic violence are summarized below. Quotes are included here to further illustrate their experiences.

### Mood or psychosomatic complaints

When asked if there were any indicators that led them to suspect domestic violence, the PCPs mentioned about patients’ mood symptoms and excessive level of stress in addition to unexplained severe bruises.

“The patient might come with multiple somatic complaints, and upon further questioning, [you might] find stress, or maybe a divorce earlier on, why? Because being beaten by the husband. Cases like this are many. But no patient would come merely and explicitly for help about domestic violence.” (Group 2, P3)

“Unless the patient has obvious bruises all over the body that makes you raise your suspicion and ask the patient. But cases like this would have gone to the A&E [Accident & Emergency Department], and might not present at our clinic. If they come to us, these cases might involve abuse and violence but relatively minor. They might not have a lot of physical injuries, and these injuries may not be visible. For the same reason, they have been suppressed for a long time and resulted in having mood problems. They would then come to us to have their mood managed.” (Group 2, P4)

The PCPs had patients who came in mainly complaining about psychosomatic or depressive symptoms. More relevant cues to domestic violence might only be revealed at the end of a consultation or subsequent consultations.

*“Normally it takes more than a few questions to obtain the answer. The patient might not tell you in a direct manner, but her visit is for things like emotional distress, or breaking down in tears. Therefore, as the doctor I would ask them directly, is there anything that has happened recently, or are there any reasons for your condition? Once being asked, the patients might start crying and say “I’m getting hit by my husband” or something similar.” (Group 2, P4).*

Most of the PCPs had the experience of listening to patients’ perspectives and helped explore available resources in the community. A PCP mentioned that he would handle the case only if the patient explicitly made “suffering from troubled relationship” the main complaint of the consultation. Appropriate management would be offered in situations where suicidal ideation was reported and relationship was known to be a major cause of distress.

### Trusting doctor-patient relationship and continuity of care

The PCPs stated that it was always difficult to get a patient to disclose such a sensitive issue unless the patient proactively complained about it. It took multiple sessions to establish rapport for the patient to trust a doctor enough to share further information. The long-term trust established with the patient and the family could facilitate the management process. PCPs in the private sector agreed that the task was more feasible for them than their counterparts due to the limited continuity of care provided in the public sector.

*“As an example, one of my patients is a very understanding person. She wouldn’t take up much of my time. She spoke to a certain point, then she would stop. And the next time she came, say 2 weeks later, she would open up herself more. Only in this way could you establish and earn the patient’s trust, then she would tell you about it, about the abuses in her family, that her husband sometimes beat her, that her kids also treated her badly. Eventually she internalized everything, resigned herself to adversity and bore everything; not making a sound, not uttering a word, not arguing with her husband.” (Group 3, P5).*

In addition, a female participant mentioned that it might be easier for women to disclose domestic violence to female PCPs, although it still took her several sessions to gain patients’ trust and to make some progress in gathering information.

*“Firstly, the patient trusts you. Because after all we are both females. She would test you for a few times; she wouldn’t start talking about it at the beginning. Every time her complaints were non-specific that you didn’t really know what she was trying to present, from headache to toe pain … Only after visiting several times, she would finally talk about relevant things. And she wouldn’t tell you all in one go, only bit by bit.” (Group 3, P2).*

### Good communication skills

Good communication skills were crucial in facilitating the patients to disclose their domestic violence issues. PCPs took time to listen and let their patients express their feelings. However, some PCPs felt they should not take sides with their patient as this might interfere with the relationship between the couple.

*“As a general practitioner, I don’t want to take sides and I also think it’s inappropriate to do so. Of course, I could have taken a side to offer help, then the patient would feel that she’s got a supporter. However, I don’t want to take sides, because I don’t understand the situation. I just let her say anything she wanted to let her ventilate … … In general, it involved many social and psychological problems that I wouldn’t be able to solve. I could only let her vent. I didn’t want the couple to stand against each other, I didn’t want her husband or children to stand against her.” (Group 3, P5).*

Instead, the PCPs preferred to maintain a neutral and non-judgmental attitude, and actively listen to their patients’ distress.

*“You have to do it very slowly, like fishing, for her [the patient] to speak out. A patient of mine was suffering; she internalized everything, her son scolded her, her daughter scolded her, her husband came back and beat her. She’s done everything. She suffered, couldn’t sleep and internalized everything. However, if I am being excessive, oversensitive or overprotective, it would likely end up problematic. The entire family would break down. Working with these patients, we are actually mediators and we have to be very careful.” (Group 3, P5).*

### Training in family therapy

With postgraduate training in family therapy, a PCP reported his shift from employing various disease models to focusing on the patient’s relationship and mood issues. In addition, with the advantage of being the family doctor, he could “try to tap into the presence of violence” and understand the perspectives of the partner as well when the patient came for a consultation. He acknowledged his actions as “bold” and admitted that his interventions might run the risk of harming the doctor-patient relationship and worsening the abuse, but he still preferred to do more for the patient.

### Patients’ safety

Despite the lack of time and specific guidelines, some PCPs stressed that it was always worthwhile taking a proactive approach to check with the patient if there was immediate threat to their safety.

*“Normally I would ask a little bit about the situation, for instance how she was beaten [by the abuser] and the level of violence, to see whether it’s serious. I would usually ask if the patient called the police or sought help from social worker to moderate.” (Group 2, P4).*

They would provide general advice and resources according to the severity of the case, such as access to referral sources. For immediate life threats, patients would be referred to A&E and the police; patients with mood problems to psychiatrists; and those with social problems to medical social workers.

*“I’ve seen one case before. The patient was already consulting a psychiatrist, and his wife had been admitted to A&E before; it’s a known case. He has been violent to his wife, involving the use of knife … For that case, the patient didn’t go to A&E on the same day he visited me. Actually … if the case was a life-threatening one, life-threatening towards the family … should ask him to go straight to A&E.” (Group 2, P5).*

Some PCPs mentioned that they ought not be the ones to call the police. They felt it should be the patient’s decision to take action. However, one PCP took the initiative to contact the police when the patient, whom he had looked after for over 10 years, came to him with severe bruises.

*“The patient was indecisive on calling the police herself, but she consulted me. I saw that she was beaten up with bruises of different colors, what else was there to say and we made the decision on her behalf.*

*It was good that she was admitted to the hospital, and the police investigated her husband’s abuse towards her, he reduced his problematic behaviors. At the end, they were living separately in two different public flats. It is hard to say for her husband’s situation, but for her and her family, it was beneficial that at the end she started a new life.” (Group 3, P3).*

### Collaboration with social workers

The majority of PCPs agreed that domestic violence was not solely a medical issue, but a social one as well. They tended to refer the cases to social workers, often due to consultation time constraints.

*“I saw an old man who came for a follow up consultation with his wife. At some point it looked like the old man wanted to hit his wife with his walking stick. When I asked her if he also acted like this at home, the old lady started crying. I referred her to our medical social worker.” (Group 2, P2).*

*“Most of the time, social workers are the ones who deal with the problem at the end. After all, our consultation time is limited, and we may not have a lot of experience, such as counselling, to deal with this kind of problems.” (Group 2, P4).*

*“When there is an injury, it has already crossed the line to determine whether to call the police. But before calling the police, I would advise them to visit a social worker first, just to see if we could keep it low key and keep the problem under control. Do we need the extra attention? Is it so serious to the point that we need the police? Or can we work out the problem with the social worker?” (Group 4, P4).*

On the other hand, the PCPs in public clinics also received referrals of domestic violence cases from social workers to manage mood problems.

*“The patient was found to have some mood problems, so s/he was advised to see the doctor. I had one such case recently … The patient came and I asked her the reason for consultation. She said she didn’t know; it was the social worker who told her to come. It took [me] a very long time to find out what it was all about. Most of the time I don’t think they [the patients] would be proactive in seeking help.” (Group 2, P4).*

Working with social workers could help manage the psychosocial issues of the victims and their partners.

*“It’s the social environment of the family that has the power to make some changes; We PCPs could work as a part of such an environment, but little we could do without the others.” (Group 4, P6).*

The PCP believed that the abuser would not remain as abuser forever once underlying personal and social problems resolved. Other examples were alcohol and gambling addictions. The curing of these problems might help alleviate or change the abusive behaviors.

The private PCPs described their encounter(s) with patients of domestic violence. Most of them had the experience of referring patients to seek help from non-governmental organizations specialized in providing social services for domestic violence victims. Among them, two female PCPs expressed that they still did not feel ready to handle cases of domestic violence. Although they understood that not all patients would contact the organizations suggested, arranging referrals to social workers and encouraging the patient to seek help seemed to be the best management they could offer. Others mentioned about their collaboration with local family support centers for patients suffering from domestic violence. They, as PCPs, were mainly expected to provide medical care for the patients’ mood problems. Psychiatrists might be invited as well for more complicated cases. They felt it was helpful to receive feedback from co-workers and empower them to provide comprehensive management.

### PCP as last resort

The PCPs felt that “actions should be taken” in domestic violence cases were possibly backed up more by a moral obligation rather than professional competence. A PCP expressed that as frontline medical professional, it was for the “greater good of society” to intervene. Some PCPs found it difficult when the patients were ambivalent whether to seek help or not. A PCP argued that if a patient had actually sought help, the doctor could at least try to understand more about the situation. Some agreed that it was common for patients to view their PCPs as the last resort.

*“I think the general practitioner is the patient’s last line of defense. If you don’t pick up those cues, and you don’t save them, where can they ask for help? They could only ask for paracetamols … … The patients may not have the knowledge. If they knew how to find a social worker by themselves, they would have done so already, or might have already solved the problem. But they do not know what to do about it.” (Group 3, P2).*

*“The victim was beaten up like a “pighead” [a Chinese description of severe physical abuse with a very swollen face]. You ought to call the police for her, right? If you just let her go home, she would end up really bad.” (Group 3, P5).*

### Questionnaire survey

There were 504 survey respondents after three rounds of questionnaire mailing from January to August 2018, with an overall response rate of 33.3% (504 /1515). Of the respondents, 59.4% were male and 40.6% were female; 49.3% were from the public and 50.7% from the private service sector. The mean (SD) years after graduation from medical school was 22.0 (12.42). Details of their background were reported elsewhere [21].

### Views on facilitators to recognition and management of domestic violence

Almost all of the survey respondents agreed/strongly agreed that a trusting doctor-patient relationship (99.8%) and good communication skills (99.0%) were facilitators to management of domestic violence (Table 2). Most also agreed that unexplained bruises of the patients (96.3%), relevant information recorded in medical history (94.6%) and mood symptoms helped to recognize patients with domestic violence (94.4%). Further, many thought that being the regular doctor of the patients (94.3%) and having a role in managing mental health problems of the patients (91.7%) were facilitators to management.

**Table 2:** Survey results on the facilitators to management of domestic violence

		Strongly disagree		Disagree		Agree		Strongly agree		Combined “strongly agree” and “agree”
		*n*	%	*n*	%	*n*	%	*n*	%	%
a	Unexplained bruises of the patients help to reveal underlying domestic violence issues	1	(0.2)	18	(3.6)	409	(81.2)	76	(15.1)	96.3%
b	Mood symptoms of the patients help to reveal underlying domestic violence issues	2	(0.4)	26	(5.2)	410	(81.3)	66	(13.1)	94.4%
c	Having a role in managing mental health problems of the patients experiencing domestic violence	2	(0.4)	40	(8.0)	415	(82.7)	45	(9.0)	91.7%
d	Being the regular doctor of the patients	0	(0.0)	29	(5.8)	339	(67.3)	136	(27.0)	94.3%
e	Looking after other members of the patients’ family	3	(0.6)	58	(11.5)	404	(80.3)	38	(7.6)	87.9%
f	Good communication skills	0	(0.0)	5	(1.0)	327	(64.9)	172	(34.1)	99.0%
g	Trusting doctor-patient relationship	0	(0.0)	1	(0.2)	279	(55.4)	224	(44.4)	99.8%
h	Relevant information recorded in patients’ medical history	0	(0.0)	27	(5.4)	390	(77.5)	86	(17.1)	94.6%
i	Known cases referred by social workers	2	(0.4)	61	(12.1)	331	(65.8)	109	(21.7)	87.5%
j	Management protocol for patient safety taught in medical training	10	(2.0)	107	(21.3)	333	(66.2)	53	(10.5)	76.7%
k	Specific training in management of domestic violence	10	(2.0)	60	(11.9)	324	(64.3)	110	(21.8)	86.1%
l	Being interested in management of domestic violence	6	(1.2)	71	(14.1)	349	(69.2)	78	(15.5)	84.7%
m	Discussion of the cases (anonymized) with colleagues	2	(0.4)	66	(13.1)	385	(76.4)	51	(10.1)	86.5%

Over 80% agreed that looking after patients’ family members (87.9%), known cases referred by social workers (87.5%), discussion of the cases with colleagues (86.5%), specific training in management of domestic violence (86.1%) and being interested in management of domestic violence (84.7%) would help. Relatively, lower percentage (76.7%) of respondents agreed with management protocol for patient safety taught in medical training as a facilitator.

### Association of PCPs’ background characteristics with facilitators

Table 3 compares the mean scores of different facilitators with the variations in PCP’s background, including the number of years in primary care practice, gender, service sector and training background. A higher mean score implied a perceived stronger facilitator to managing domestic violence. Significant differences (*P* < 0.05) were indicated by the results of ANOVA. PCPs who had practiced more than 20 years were more likely to perceive unexplained bruises and patients’ medical history as facilitators. Those with Fellowship from RACGP, RCGP or HKCFP, or master/diploma in psychological medicine or (family) counseling, or medical degree from western countries, they were all more likely to regard patients’ medical history as a facilitator. PCPs with more than 10 years’ experience, or those in private practice, were more likely to perceive looking after other members of the patients’ family as a facilitator. Male PCPs tended to perceive good communication skills and being interested in the management of domestic violence as strong facilitators.

**Table 3:** Association of PCPs’ background characteristics with mean scores of different facilitators to managing domestic violence

	(a) Unexplained bruises	(b) Mood symptoms	(c) Role in mental health management	(d) Regular doctor	(e) Other family members	(f)Communi-cation skills	(g) Trusting relationship	(h) Medical history	(i) Referred by social workers	(j) Safety protocol	(k) Specific training	(l) Interested in DV management	(m)Discus-sion with colleague
**No. of years in primary care practice**													
< 10	3.08	3.06	2.99	3.13	2.86	3.31	3.39	3.08	3.14	2.87	3.10	2.97	3.06
10–19	3.07	3.06	3.01	3.21	2.95	3.31	3.44	3.04	3.09	2.87	3.09	2.96	2.94
20–29	3.17	3.08	2.97	3.24	2.93	3.37	3.50	3.21	3.05	2.76	3.03	3.04	2.90
≥ 30	3.12	3.07	3.01	3.21	2.94	3.33	3.44	3.11	3.00	2.92	2.96	3.01	2.95
P-value^a^	0.032*	0.763	0.600	0.255	0.015*	0.634	0.499	0.008*	0.381	0.336	0.391	0.691	0.106
**Gender**													
M	3.14	3.07	2.99	3.24	2.96	3.37	3.48	3.14	3.08	2.88	3.09	3.05	2.96
F	3.07	3.07	3.02	3.17	2.94	3.28	3.40	3.08	3.10	2.82	3.00	2.91	2.97
*P*-value	0.100	0.937	0.333	0.150	0.626	0.029*	0.083	0.167	0.747	0.298	0.128	0.008*	0.755
**Setting**													
Public	3.08	3.09	3.01	3.18	2.90	3.33	3.40	3.10	3.18	2.85	3.11	3.02	2.98
Private	3.14	3.06	2.99	3.25	3.00	3.33	3.49	3.13	3.00	2.85	3.00	2.96	2.94
*P*-value	0.109	0.510	0.533	0.166	0.018*	0.976	0.061	0.417	0.001**	0.936	0.075	0.318	0.333
**Specialist in family medicine**													
Yes	3.12	3.11	3.03	3.26	2.94	3.39	3.48	3.12	3.13	2.84	3.04	3.00	2.96
No	3.10	3.05	2.99	3.18	2.95	3.30	3.42	3.12	3.06	2.86	3.06	2.98	2.96
*P*-value	0.709	0.135	0.320	0.121	0.780	0.065	0.187	0.968	0.202	0.803	0.738	0.726	0.944
**FRACGP/MRCGP/FHKCFP**^**b**^													
Yes	3.09	3.08	3.02	3.26	2.95	3.33	3.47	3.17	3.12	2.84	3.07	3.00	3.00
No	3.13	3.06	2.98	3.18	2.95	3.33	3.42	3.08	3.06	2.87	3.05	2.97	2.93
*P*-value	0.392	0.473	0.296	0.103	0.981	0.885	0.345	0.036*	0.285	0.593	0.697	0.566	0.080
**Master/Diploma in Psychological Medicine/ Counselling/Family Counselling**													
Yes	3.17	3.21	3.13	3.34	3.04	3.36	3.53	3.26	3.21	2.89	3.19	3.06	3.02
No	3.10	3.06	2.99	3.20	2.94	3.33	3.44	3.10	3.07	2.85	3.04	2.98	2.95
*P*-value	0.312	0.019*	0.036*	0.084	0.141	0.671	0.212	0.028*	0.123	0.622	0.130	0.350	0.375
**1st Medical degree**													
Hong Kong	3.11	3.08	2.99	3.18	2.94	3.31	3.41	3.09	3.11	2.85	3.07	2.98	2.95
Other Asian countries	3.14	3.03	3.03	2.97	2.93	3.21	3.28	3.07	2.90	2.83	2.93	2.90	2.93
Western countries	3.13	3.06	3.05	3.46	3.01	3.50	3.67	3.30	3.05	2.87	3.04	3.06	3.01
*P*-value	0.873	0.873	0.476	< 0.001**	0.402	0.003*	< 0.001**	0.001*	0.145	0.943	0.507	0.358	0.602

*P < 0.05; **P < 0.001

^a^*P*-value of ANOVA

^b^FRACGP: Fellowship of the Royal Australian College of General Practitioners; MRCGP: Membership of the Royal College of General Practitioners; FHKCFP: Fellowship of Hong Kong College of Family Physicians

Apart from patients’ medical history, PCPs with higher training in mental health or counselling were also more likely to regard cues from mood symptoms of the patients, and their role in managing patients’ mental health problems as facilitators. Whereas PCPs who graduated from western countries would give more weight to being the regular doctor of the patient, good communication skills, and a trusting doctor-patient relationship as facilitators to the management of domestic violence.

## Discussion

The study found that both mood problems and unexplained bruises were useful indicators for recognizing victims of domestic violence. Most of the survey respondents believed that mood symptoms would help identify victims. Stronger facilitators were perceived by PCPs with postgraduate training in psychological medicine, or (family) counselling. These mood problems included psychosomatic or depressive symptoms as well as suicidal ideations. Having relevant information recorded in patient’s medical history was identified as another strong facilitator for recognizing and managing victims of domestic violence. It is important that PCPs are able to recognize these indicators at an early stage to prevent the abuse from worsening.

Without a screening policy and local training protocol for domestic violence management, it is only when patients are faced with life-threatening emergencies would PCPs in Hong Kong seek intervention from the police. In our survey, 76.6% of respondents regarded the management protocol for patient safety taught during their medical training as a facilitator. Although this was the least identified facilitator in the current study, foreign experiences show that the development of a specific protocol for managing victims of domestic violence would greatly facilitate healthcare professionals to help these patients. A study in Malaysia suggested that a lack of positive attitude and confidence among healthcare workers for the identification and management of domestic violence might have resulted from inadequate knowledge to do so [25]. A U.S. study found that even physicians had a sense of responsibility to assist the victims, many believed that they would not have access to suitable resources [26]. International experiences showed that physician training combined with system support is important for facilitating PCPs to help these victims [27].

Almost all of our survey respondents agreed that a good doctor-patient relationship was fundamental for the management of patients who experienced domestic violence. However, just as highlighted in our earlier study, public clinic attenders perceived stronger barriers in seeking help for psychological distress was due to not having a regular PCP [28]. PCPs in the public sector often failed to establish a trusting relationship with their patients as continuity of care was not possible. Interviewees pointed out that it often took multiple consultations before patients were willing to express their concerns over being abused. On the contrary, it was far more feasible for private PCPs to establish a strong relationship with their patients who were more likely to see them for multiple consultations with less time constraints. Our previous study had identified that patients with a regular PCP had a higher likelihood of receiving mental healthcare [28]. Furthermore, 99% of our survey respondents in the current study believed that having good communication skills was crucial to enable better management of victims. Interviewees also mentioned that active listening with a non-judgmental attitude helped relieve their patient’s distress.

Doctors with postgraduate training in family therapy or psychological medicine displayed a more proactive attitude when managing patients whom they suspected were victims of domestic violence. In fact, a U.K. study found that some patients would expect health professionals to take the initiative to ask about domestic abuse, especially males [29]. U.S. studies also suggested that PCPs with specific training in domestic violence and female physicians in particular felt more comfortable addressing domestic abuse [30]. Physicians in the U.S. who received domestic violence education were also significantly more likely to screen for domestic violence [31]. Our study recruited PCPs who had the advantage of taking care of all members of a family unit, which allowed them the opportunity to understand the situation from the partner’s point of view as well as that of the patient. This was more evident in the private sector as shown in the results.

While U.S. studies had looked into how nurses and PCPs both contributed in the management of patients experiencing domestic violence [19], our interviewees emphasized the role of social workers in a multidisciplinary medical-social collaboration. In Hong Kong, according to the Domestic Violence Ordinance (Cap. 189) enacted in 1986, social workers are expected to be in charge, despite the lack of established protocol, of responding to a suspected case, coordinating with different professionals and arranging interventions and even mediation if needed [32, 33]. Our interviewees mentioned that a deprived social-economic environment was often the root cause for domestic violence, which relied on the intervention of social workers as doctors had limited involvement in patients’ social lives. They thought it was also crucial to maintain a good relationship with social workers to handle psychosocial issues of the patients. In addition, over 87% of our survey respondents believed that cases referred by social workers would be a strong facilitator.

Apart from family therapy and psychological medicine courses, it is recommended that specific training programs for the management of domestic violence victims be provided to PCPs to enhance their competence in dealing with these patients. When designing these programs, it is essential to adopt culturally appropriate intervention models suitable for Chinese victims [34]. One of the possible ways to do so is by incorporating interactive simulation-based programs to enhance cultural and language acceptability and transferability across communities. For instance, an interactive simulation-based program developed in Australia have shown a significant impact in teaching healthcare workers in Hong Kong about patient deterioration management [35]. It may also be an effective program in educating healthcare workers on the management of domestic violence victims.

This study had some limitations. First, the response rate of the survey was 33.3% which could not be considered good for this kind of study, but is higher than most other surveys among doctors in Hong Kong [36, 37]. Second, the proportion of survey respondents from the public sector (49.3%) was higher than their corresponding proportion (about 25%) in Hong Kong. Although we intended to have proportionate representation in the survey, the half-half proportions of PCP respondents from the public and private sectors might be an advantage for us to compare their responses.

## Conclusions

Our findings illustrate several major facilitators for PCPs to recognize and manage domestic violence in Hong Kong, despite the lack of a screening policy and specific training protocols. Moreover, they also have a global impact in that the facilitators could provide the basis for ameliorating, comparing and evaluating the training for PCPs in early detection of domestic violence across diverse geographical and cultural settings. Mood symptoms are strong indicators for probable abuse, while communication skills and continuity of care are important to unmask issues of domestic violence. Training programs in family counselling or psychological medicine can help improve the knowledge and confidence of PCPs in dealing with suspected domestic violence cases. In addition, cooperation with social workers is important for managing psychosocial issues of the patients and their partners. A comprehensive protocol emphasizing medical-social collaboration is highly valued by PCPs to facilitate them to take a more proactive and effective stance from screening to managing domestic violence with multidisciplinary teams. These facilitators are particularly important to be targeted in China and other Asian countries through policy implementation and continuing medical education.

## Supplementary information

**Additional file 1.** : Supplementary file – relevant sections from the questionnaire

## Data Availability

The datasets used and/or analyzed during the current study are available from the corresponding author on reasonable request.

## Abbreviations

PCP: Primary Care Physician
HKCFP: Hong Kong College of Family Physicians
A&E: Accident & Emergency Department
FRACGP: The Fellowship of the Royal Australian College of General Practitioners
MRCGP: Membership of the Royal College of General Practitioners
FHKCFP: Fellow, Hong Kong College of Family Physicians
