# Supplementary material for: Factors that facilitate recognition and management of domestic violence by primary care physicians in a Chinese context - a mixed methods study in Hong Kong
Source: BMC Fam Pract. 2020 Jul 30;21:155. doi: 10.1186/s12875-020-01228-4 (PMC7394675; doi:10.1186/s12875-020-01228-4)
Supplement: Supplementary file 1 — Additional file 1. : Supplementary file – relevant sections from the questionnaire [file 12875_2020_1228_MOESM1_ESM.docx]

Supplementary file – relevant sections from the questionnaire

Study on family physicians’ facilitators to management of domestic violence

| **DEFINITIONS: Domestic violence, also known as intimate partner violence, includes any behavior causing physical, emotional and/or sexual harm to the adult partner. *(This survey excludes child abuse.)***  **Management of domestic violence includes detection, management and referral of patients experiencing domestic violence** |
| --- |

*Please tick the most appropriate box for the following items*

| **Are the followings your facilitators to management of domestic violence?** | | | strongly disagree | | disagree | | agree | | strongly agree |
| --- | --- | --- | --- | --- | --- | --- | --- | --- | --- |
|  |  | | 1 | | 2 | | 3 | | 4 |
| a. | Unexplained bruises of the patients help to reveal underlying domestic violence issues | | □ | | □ | | □ | | □ |
| b. | Mood symptoms of the patients help to reveal underlying domestic violence issues | | □ | | □ | | □ | | □ |
| c. | Having a role in managing mental health problems of the patients experiencing domestic violence | | □ | | □ | | □ | | □ |
| d. | Being the regular doctor of the patients | | □ | | □ | | □ | | □ |
| e. | | Looking after other members of the patients’ family | | □ | | □ | | □ | □ |
| f. | | Good communication skills | | □ | | □ | | □ | □ |
| g. | | Trusting doctor-patient relationship | | □ | | □ | | □ | □ |
| h. | | Relevant information recorded in patients’ medical history | | □ | | □ | | □ | □ |
| i. | | Known cases referred by social workers | | □ | | □ | | □ | □ |
| j. | | Management protocol for patient safety taught in medical training | | □ | | □ | | □ | □ |
| k. | | Specific training in management of domestic violence | | □ | | □ | | □ | □ |
| l. | | Being interested in management of domestic violence | | □ | | □ | | □ | □ |
| m. | | Discussion of the cases (anonymized) with colleagues | | □ | | □ | | □ | □ |

| **Please provide some information about yourself which will remain strictly confidential** | | |
| --- | --- | --- |
| a. | Your year of graduation from medical school | ⬜⬜⬜⬜ *(yyyy)* |
| b. | Your number of years in primary care practice | ⬜⬜ |
| c. | Your sex is | ⬜ Male ⬜ Female |
| d. | You work mainly in | ⬜ Hospital ⬜ Community |
| e. | Your practice is | ⬜ Public ⬜ Private |
| f. | Your practice is | ⬜ Solo ⬜ Group |
| g. | You obtained your first medical degree in: | |
|  | ⬜ Hong Kong ⬜ Mainland ⬜ Australasia ⬜ Canada ⬜ UK ⬜ US Others:_________________ | |
| h. | Your higher qualifications: (can choose more than one item if applicable) | |
|  | ⬜ Specialist in Family Medicine ⬜ FRACGP/MRCGP/FHKCFP ⬜ Diploma in Family Medicine | |
|  | ⬜ PDip in Community Psychological Medicine (HKU) ⬜ Master in Family Counselling/Therapy | |
|  | ⬜ Master/Diploma in Counselling ⬜ Others:________________________________ ⬜ NA | |
